# Supplementary material for: Spatial alanine metabolism determines local growth dynamics of Escherichia coli colonies
Source: eLife. 2021 Nov 9;10:e70794. doi: 10.7554/eLife.70794 (PMC8579308; doi:10.7554/eLife.70794)
Supplement: Supplementary file 1. — Abbreviations: Kan = kanamycin. Superscript “R” = resistance. “-” = fusion. “::” = insertion. The scar corresponds to 5’-GAAGTTCCTATACTTTCTAGAGAATAGGAACTTC-3’ sequence. [file elife-70794-supp1.docx]

**Supplementary File 1: Bacterial strains used in this study.** Abbreviations: Kan = kanamycin. Superscript “R” = resistance. “-” = fusion. “::” = insertion. The scar corresponds to the sequence 5’-GAAGTTCCTATACTTTCTAGAGAATAGGAACTTC-3’.

| **Strain** | **Genotype/ Relevant features** | **Reference** |
| --- | --- | --- |
| KDE261 | *E. coli* strain carrying plasmid pCP20. | Drescher lab stock |
| KDE262 | *E. coli* strain carrying plasmid pKD46. | Drescher lab stock |
| KDE264 | *E. coli* strain carrying plasmid pKD3. | Drescher lab stock |
| KDE265 | *E. coli* strain carrying plasmid pKD4. | Drescher lab stock |
| KDE1361 | *E. coli* strain carrying plasmid pNUT1361. | Drescher lab stock |
| KDE2338 | *E. coli* strain carrying plasmid pNUT2338. | Drescher lab stock |
| KDE2658 | *E. coli* strain carrying plasmid pUC18R6KT-mini-Tn7-Km (Addgene #64969). | Drescher lab stock |
| KDE2659 | *E. coli* strain carrying plasmid pTNS2 (Addgene #64968). | Drescher lab stock |
| KDE2674 | *E. coli* strain carrying plasmid pNUT2674. | This study |
| KDE2787 | *E. coli* strain carrying plasmid pNUT2787. | This study |
| KDE2838 | *E. coli* strain carrying plasmid pNUT2838. | This study |
| KDE474 | *E. coli* AR3110 WT. | (Serra, Richter, & Hengge, 2013) |
| KDE679 | AR3110, P*_tac_-mRuby2-mRuby2* and *Kan^R^* inserted at *attB* site (P*_tac_* without operator). | (Vidakovic, Singh, Hartmann, Nadell, & Drescher, 2018) |
| KDE722 | KDE679 with Δ*fliC*::scar. | (Vidakovic et al., 2018) |
| KDE1899 | AR3110 with Δ*fliC*::scar. | This study |
| KDE2007 | KDE679 with Δ*fliC*::scar, Δ*alaE*::scar. | This study |
| KDE2009 | KDE679 with Δ*fliC*::scar, Δ*dadAX*::scar. | This study |
| KDE2086 | KDE679 with Δ*fliC*::scar, Δ*alaE*::scar, Δ*dadAX*::scar. | This study |
| KDE2183 | KDE679 with Δ*fliC*::scar, Δ*cycA*::scar. | This study |
| KDE2185 | KDE679 with Δ*fliC*::scar, Δ*livG*::scar. | This study |
| KDE2438 | KDE679 with Δ*fliC*::scar, Δ*yaaJ*::scar. | This study |
| KDE2242 | AR3110, P*_tac_-sfgfp-sfgfp* and *Kan^R^* inserted at *attB* site (P*_tac_* without operator), with Δ*fliC*::scar, Δ*alaE*::scar, Δ*dadAX*::scar. | This study |
| KDE2445 | AR3110, P*_tac_-sfgfp-sfgfp* and *Kan^R^* inserted at *attB* site (P*_tac_* without operator), with Δ*fliC*::scar. | This study |
| KDE2533 | KDE679 with Δ*fliC*::scar Δ*cycA*::scar, Δ*livG*::scar, Δ*alaE*::scar, Δ*yaaJ*::scar. | This study |
| KDE2564 | KDE679 with Δ*fliC*::scar, Δ*cycA*::scar, Δ*dadAX*::scar. | This study |
| KDE2607 | KDE679 with Δ*fliC*::scar, Δ*yaaJ*::scar, Δ*dadAX*::scar. | This study |
| KDE2937 | KDE679 with Δ*fliC*::scar, P*_tac_-sfgfp*(ASV) at the Tn7 insertion site, coding for an unstable superfolder GFP with an AANDENYAASV-tag. | This study |
| KDE2938 | KDE679 with Δ*fliC*::scar, Δ*alaE*::scar, P*_tac_-sfgfp*(ASV) at the Tn7 insertion site. | This study |
| KDE2939 | KDE679 with Δ*fliC*::scar, Δ*dadAX*::scar, P*_tac_-sfgfp*(ASV) at the Tn7 insertion site. | This study |
| KDE2940 | KDE679 with Δ*fliC*::scar, Δ*alaE* Δ*dadAX*, P*_tac_-sfgfp*(ASV) at the Tn7 insertion site. | This study |
