# Supplementary material for: Spatial alanine metabolism determines local growth dynamics of Escherichia coli colonies
Source: eLife. 2021 Nov 9;10:e70794. doi: 10.7554/eLife.70794 (PMC8579308; doi:10.7554/eLife.70794)
Supplement: Supplementary file 2. — Abbreviations: Kan = kanamycin, Amp = ampicillin, Chl = chloramphenicol. Superscript “R” = resistance. “-“ = fusion. [file elife-70794-supp2.docx]

**Supplementary File 2: Plasmids used in this study.** Abbreviations: Kan = kanamycin, Amp = ampicillin, Chl = chloramphenicol. Superscript “R” = resistance. “-“ = fusion.

| **Plasmid** | **Genotype/ Relevant features** | **Reference** |
| --- | --- | --- |
| pKD3 | R6K ori, chloramphenicol acetyl transferase cassette flanked *by frt*, Amp^R^. | (Datsenko & Wanner, 2000) |
| pKD4 | R6K ori, *T*n5 neomycin phosphotransferase cassette flanked *by frt*, Amp^R^. | (Datsenko & Wanner, 2000) |
| pKD46 | Temperature sensitive replication origin oriR101, arabinose-inducible lambda recombinase genes*,* Amp^R^. | (Datsenko & Wanner, 2000) |
| pCP20 | Temperature sensitive replication origin oriR101, Flp recombinase gene, Chl^R^, Amp^R^. | (Cherepanov & Wackernagel, 1995) |
| pNUT1361 | pSC101*, Kan***^R^,*** P*_tac_-sfgfp*. | Drescher lab stock |
| pNUT2338 | pSC101*, Kan***^R^,*** P*_tac_-mRuby2-mRuby2*. | This study |
| pUC18R6KT-mini-Tn7-Km | R6K ori, contains *Tn7L* and *Tn7R* sites, between these sites a *T*n5 neomycin phosphotransferase cassette flanked *by frt*, Amp^R^. | (Choi et al., 2005), Addgene #64969 |
| pTNS2 | R6K ori, plasmid for transposase (*tnsABCD*) expression, Amp^R^. | (Choi et al., 2005), Addgene #64968 |
| pNUT2674 | pKD4 backbone, P*_tac_-sfgfp*(ASV), coding for an unstable superfolder GFP with the AANDENYAASV-tag. | This study |
| pNUT2787 | pUC18R6KT-mini-Tn7-Km backbone. Tn5 neomycin phosphotransferase cassette replaced by a chloramphenicol acetyl transferase cassette | This study |
| pNUT2838 | pNUT2787 backbone. P*_tac_-sfgfp*(ASV), coding for an unstable superfolder GFP with the AANDENYAASV-tag, placed between *Tn7L* and *Tn7R* sites. | This study |
