# Supplementary material for: Spatial alanine metabolism determines local growth dynamics of Escherichia coli colonies
Source: eLife. 2021 Nov 9;10:e70794. doi: 10.7554/eLife.70794 (PMC8579308; doi:10.7554/eLife.70794)
Supplement: Supplementary file 3. [file elife-70794-supp3.docx]

**Supplementary File 3: DNA oligonucleotides used in this study.**

| **Name** | **Sequence (5’ to 3’ direction)** | **Description** |
| --- | --- | --- |
| KDO834 | ACAACTTTTTGTCTTTTTACCTTCCCGTTTCGCTCAAGTTAGTATTTGACAATTAATCATCGGCTCGTATAATG | Insertions at the *attB* site |
| KDO894 | TGGCTGTTTTTGAAAAAAATTCTAAAGGTTGTTTTACGACGTGTAGGCTGGAGCTGCTTC | *fliC* deletion |
| KDO895 | AATCAGGTTACAACGATTAACCCTGCAGCAGAGACAGAACCTGCATATGAATATCCTCCTTAG | *fliC* deletion |
| KDO1662 | TCCGGGCTATGAAATAGAAAAATGAATCCGTTGAAGCCTGCTTTTCATGGGAATTAGCCATGGTCC | Insertions at the *attB* site |
| KDO2562 | CATCTCCATTAACATCCCATTACGCTTTTATTAAGGAGCATTAGCGTGTAGGCTGGAGCTGCTTC | *alaE* deletion |
| KDO2563 | GCCAGTTAAAGACGCGACTGGCGATGCCAGTCGCGAAAAGAAGAGATGGGAATTAGCCATGGTCC | *alaE* deletion |
| KDO2566 | TTAGATTATTATTCTTTTACTGTATCTACCGTTATCGGAGTGGCTGTGTAGGCTGGAGCTGCTTC | *dadAX* deletion |
| KDO2567 | TTTTTGCACCCAGAAGACGTTGCCTCCGATCCGGCTTACAACAAGATGGGAATTAGCCATGGTCC | *dadAX* deletion |
| KDO2845 | CGTAGAGCCTGAACAACACAGACAGGTACAGGAAGAAAAAAACGTGTAGGCTGGAGCTGCTTC | *cycA* deletion |
| KDO2846 | CTAAAAGCTGGATGGCATTGCGCCATCCAGCATGATAATGCGGGGTCCATATGAATATCCTCCTTAG | *cycA* deletion |
| KDO2841 | ACTGAAGCTGAAAAACGGCGCAGCGAAAGGAGAGCAGGCATGATTGTGTAGGCTGGAGCTGCTTC | *livG* deletion |
| KDO2842 | GGCGCTGACTTTGTCAAAGGACAACATGACTTTTTCCATCTTAGGTCCATATGAATATCCTCCTTAG | *livG* deletion |
| KDO3481 | GTTTACACAGGAAAGTCATCGCGACCGGCAATAAGAGGGATATGCGTGTAGGCTGGAGCTGCTTC | *yaaJ* deletion |
| KDO3482 | GCCGACTTTAGCAAAAAATGAGAATGAGTTGATCGATAGTTGTGAATGGGAATTAGCCATGGTCCAT | *yaaJ* deletion |
| KDO3256 | ACCTTTGCTAACCATCAACCACCTCCTTTAGTTTAATTAAGGTG | pNUT2338 construction |
| KDO3257 | GTACCGCTAGCGGTGTAGGCTGGAGCTGCTTC | pNUT2338 construction |
| KDO3817 | TCGACTCCTTCTCGAGGAATTCCTGCAGCC | pNUT2838 construction |
| KDO3818 | GAATGCTGGTACCTCGCGAAGGCCT | pNUT2838 construction |
| KDO3785 | TAGTTTTCATCGTTCGCGGCTTTATACAGTTCATCCATGCCGTGG | pNUT2674 construction |
| KDO3786 | GCCGCGAACGATGAAAACTATGCCGCGAGCGTGTAAAGACATGA | pNUT2674 construction |
| KDO4121 | CGCTTGGACGGAAGAGTATGAGGATCCAACATTTC | pNUT2787 construction |
| KDO4121 | CCAGATATCCGATCCTCATCCTGTCTCTTG | pNUT2787 construction |
| KDO4127 | CGCGAGGTACCAGCATTCGCTTGGATTCTCACCAATGG | pNUT2838 construction |
